# Amplified Spontaneous Emission and Carrier Dynamics in Ruddlesden–Popper and Dion–Jacobson Quasi‐2D Perovskite

**DOI:** 10.1002/nap2.70335

**Published:** 2026-11-10

**Authors:** Chaochao Qin, Xinyu Zhang, Minghuan Cui, Haiying Wang, Pan Song, Jian Song, Shuhong Ma, Zhaoyong Jiao, Guangrui Jia, Yuhai Jiang, Zhongpo Zhou

**Affiliations:** ^1^ Henan Province Engineering Research Center of Optoelectronic Technology and Advanced Manufacturing, and School of Physics Henan Normal University Xinxiang China

**Keywords:** amplified spontaneous emission, exciton‐phonon coupling, quasi‐2D perovskites, threshold, transient absorption

## Abstract

Ruddlesden–Popper (RP) and Dion–Jacobson (DJ) quasi‐2D perovskites exhibit disparate chemical bonds and structures due to distinct organic spacer cations, endowing them with distinctive physical properties. A more far‐reaching investigation of the fundamental structure–physical relation, including the carrier dynamics, is expected in these layered perovskites. The phase distribution, defect state density, and ultrafast dynamics of RP‐type perovskite (PA)_2_ (${\text{FA}}_{0.8}{\text{Cs}}_{0.2}$)_3_ ${\text{Pb}}_{4}{\text{Br}}_{13}$ and DJ‐type perovskite (HDAD) (${\text{FA}}_{0.8}{\text{Cs}}_{0.2}$)_3_ ${\text{Pb}}_{4}{\text{Br}}_{13}$ were investigated by using transient absorption spectra. The DJ‐type perovskite exhibits a lower threshold amplified spontaneous emission (ASE); however, its Auger recombination is more severe than that of RP‐type perovskite. By adding a trace amount of HBr to the solvent during crystal growth, exciton–phonon coupling was inhibited, energy transfer was promoted, and the Auger recombination was suppressed. We ultimately chose the HBr‐treated HDAD quasi‐2D perovskite thin films, achieving a low ASE threshold and high‐performance single‐mode green lasers.

## Graphical abstract

Ruddlesden–Popper and Dion–Jacobson quasi‐2D perovskites exhibit disparate chemical bonds and structures due to distinct organic spacer cations, endowing them with distinctive physical properties. A far‐reaching investigation of the structure–physical relation, including the carrier dynamics, is expected in these layered perovskites. We ultimately chose the HBr‐treated HDAD Dion–Jacobson perovskite thin films, achieving a low ASE threshold and high‐performance single‐mode green lasers.

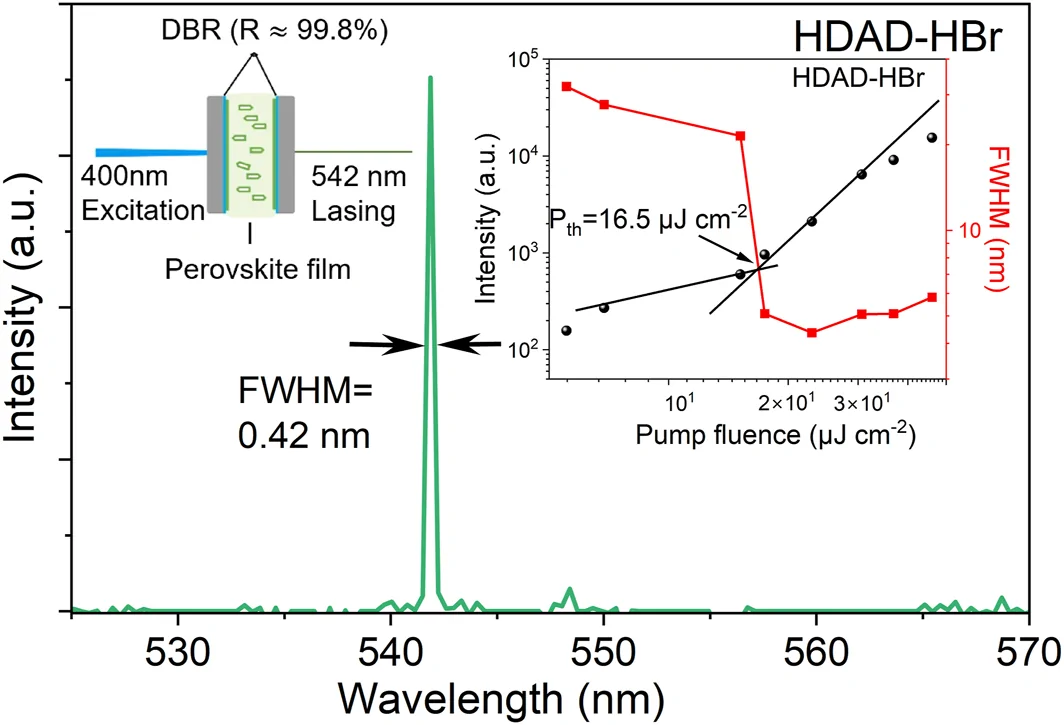

## 1 Introduction

Organic–inorganic halide perovskite semiconductors have garnered intense research interest for light‐emitting applications due to their superior properties, including tunable bandgap [1], narrow‐bandwidth light emission, and high photoluminescence quantum yield (PLQY) [2]. Their low‐threshold and wavelength‐tunable amplified spontaneous emission (ASE) through stoichiometric adjustment and compositional regulation renders them ideal candidates for state‐of‐the‐art research into next‐generation light‐emitting devices such as light‐emitting diodes (LEDs) and lasers [3, 4]. However, the poor stability in the ambient environment hinders development toward practical implementation. One strategy to address this stability challenge is to incorporate bulky organic molecules into the perovskite framework, forming the two‐dimensional (2D) or quasi‐2D perovskites [5].

There are two typical quasi‐2D perovskites, the Ruddlesden–Popper (RP) and Dion–Jacobson (DJ) phases, defined by formulas $E_{2}A_{n-1}B_{n}X_{3n+1}$ and ${\text{FA}}_{n-1}B_{n}X_{3n+1}$, where *E* and *F* are monovalent and divalent organic spacer cations such as propylammonium [${\left(C_{3}H_{7}{\text{NH}}_{3}\right)}^{+}$, PA] and hexamethylenediammonium [H_3_N^+^(CH_2_)_6_ ${\text{NH}}_{3}^{+}$, HDAD], respectively. A is Cs^+^ or a small organic cation, such as formamidinium [HC${\left(\text{NH}\right)}_{2}^{2+}$, FA], B is Pb^2+^, *X* is a halide ion, such as Br^−^, and n is the number of layers of inorganic octahedra between adjacent organic layers [6, 7]. The RP‐type perovskites employ double monovalent ligands to separate adjacent inorganic perovskite layer units, with a van der Waals gap forming in between [8]. In contrast, the DJ‐type perovskites incorporate only one divalent spacer molecule to link adjacent inorganic octahedral layers (inset in Figure 1a). Although the inorganic octahedral layer governs core optoelectronic properties, including the bandgap and mobility, the organic spacer cations exert significant influence on physical properties, such as exciton binding energy, which is crucial for optoelectronic performance [9, 10, 11]. The energy transfer rate from small‐n to large‐n quantum wells play a vital role in achieving low‐threshold ASE, which is influenced by the population distribution among different n‐phases, defect state density, electron–phonon coupling, and Auger recombination rate [12, 13]. Although quasi‐2D perovskite lasers have achieved remarkable advances in research over recent years, the underlying factors driving these improvements remain ambiguous [14, 15, 16]. Specifically, it is unknown whether the enhancements are attributed to the organic spacer type and size [17], associated with the organic spacer's dielectric characteristics [18], caused by the differing van der Waals gap in the two phases [19], adjusted by various quantum well distributions, or mediated by other parameters. A comprehensive comparison of the two types of quasi‐2D perovskites ought to be performed by employing organic spacers with similar molecular sizes, which serves to minimize the structural discrepancies caused by variations in the length or conformation of the organic spacers.

**FIGURE 1:**
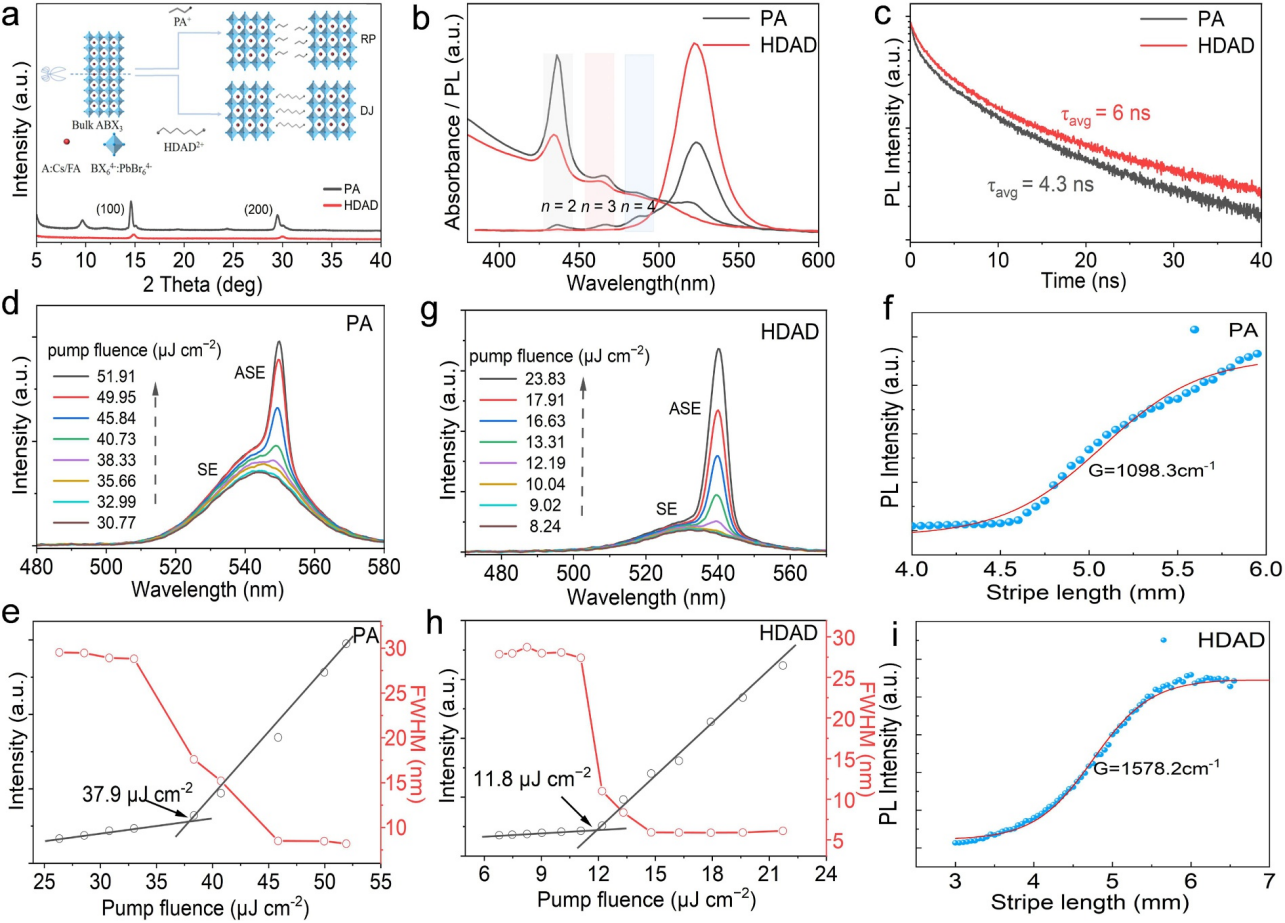
Structure and optical properties of the PA and HDAD thin films. (a) XRD patterns. (Inset: Structures of DJ‐ and RP‐type perovskites) (b) Absorption and PL spectra. (c) TRPL decay profiles. (d, g) Power‐dependent PL spectra. (e, h) PL intensity and FWHM as functions of pump intensity. (f, i) Gain coefficients.

Here, we studied the phase distribution, defect state density, electron–phonon coupling, Auger recombination rate, and ultrafast dynamics of RP and DJ perovskites by using transient absorption (TA), PLQY, and temperature‐dependent PL measurements. The (HDAD) (${\text{FA}}_{0.8}{\text{Cs}}_{0.2}$)_3_ ${\text{Pb}}_{4}{\text{Br}}_{13}$ (HDAD) perovskites exhibited lower defect state density, more favorable phase distribution, stronger PL intensity, weaker electron–phonon coupling, and a lower ASE threshold than that of the (PA)_2_ (${\text{FA}}_{0.8}{\text{Cs}}_{0.2}$)_3_ ${\text{Pb}}_{4}{\text{Br}}_{13}$ (PA) perovskite thin films. A low ASE threshold of 5.5 μJ ${\text{cm}}^{-2}$ was achieved for the improved HDAD perovskites by adding hydrobromic acid (HBr) during crystal growth, which ameliorated the Auger recombination in the films. A single‐mode laser based on the HDAD perovskite with hydrobromic acid (HDAD‐HBr) was fabricated, with a threshold of 16.5 μJ ${\text{cm}}^{-2}$.

## 2 Results and Discussion

The XRD peaks of PA and HDAD thin films observed around 15° and 30° (Figure 1a) correspond to the (100) and (200) crystal planes of the 3D phases, respectively [20]. In the PA film, diffraction peaks at 10° and 24.8° are attributed to the n=2 phase, whereas no diffraction peaks from the small‐n‐phase are detected in the HDAD film. The PA film exhibits well‐resolved peaks near 5° and 10°, assigned to the (020) and (060) planes of the n=2 phase (Supporting Information S1: Figure S1), whereas the HDAD film displays no obvious low‐angle XRD (SAXRD) peaks, confirming abundant lower‐n phases in the PA film and predominantly higher‐n phases in the HDAD film. The PA film exhibits distinct absorption peaks at 434 nm (n=2), 464 nm (n=3), 482 nm (n=4), and 523 nm (n≥5); however, the HDAD film exhibits weak absorption features, particularly the 434 nm (n=2) peak, whose intensity is one‐third that of the PA film's counterpart (Figure 1b). The PA film displays a relatively weak PL signal peaked at 435 nm (n=2), 465 nm (n=3), 485 nm (n=4), and 525 nm (n≥5). However, the HDAD film features a single dominant and intense emission peak around 525 nm (n≥5), implying prominent radiative recombination. The HDAD film possesses an average time‐resolved photoluminescence (TRPL) lifetime of 6.0 ns, which is longer than the 4.3 ns observed for the PA film (Figure 1c). The fitting parameters from the bi‐exponential function are provided in Supporting Information S1: Table S1. The prolonged PL lifetime indicates suppressed trap‐mediated nonradiative recombination in the HDAD film, which exhibits good crystallinity and a highly homogeneous phase distribution. The PLQY of PA film and HDAD film is 19% and 26.5%, respectively (Supporting Information S1: Figure S2), implying that the PA films suffer from more pronounced defect‐induced nonradiative recombination.

The PA film exhibits a spontaneous emission (SE) peak centered at 544 nm with a full width at half‐maximum (FWHM) of 30 nm at a pump fluence of 30.77 μJ ${\text{cm}}^{-2}$ (Figure 1d). When the pump fluence exceeds a threshold $\left(P_{th}\right)$ of 35.66 μJ ${\text{cm}}^{-2}$, a narrow peak emerges on the low‐energy side of the SE, centered at 550 nm with a FWHM of 17.4 nm. In addition, a distinct linear‐to‐superlinear transition in PL intensity and a marked narrowing of the FWHM near the threshold confirm the onset of ASE, with a threshold of 37.9 μJ ${\text{cm}}^{-2}$ for the PA film (Figure 1e). Similarly, the HDAD film shows SE and ASE peaks around 528 and 540 nm, respectively, with a lower ASE threshold of 11.8 μJ ${\text{cm}}^{-2}$ (Figure 1g,h). In addition to the ASE threshold, the net modal gain coefficient (G) is a key performance metric for the optical amplification capabilities, which is usually measured using the variable stripe length (VSL) method [21]. The output intensity $I_{ASE}$ was collected at each varying excited stripe length (L), $I_{ASE}=I_{0}Ae^{GL}-1/G$, where $I_{0}$ is the pump energy intensity, and A is a constant related to the emission cross‐section. The G values were determined to be 1098.3 ${\text{cm}}^{-1}$ and 1578.2 ${\text{cm}}^{-1}$ at a pump power of 15.24 mW for the PA film and the HDAD film, respectively (Figure 1f–i). The low ASE threshold and large gain coefficient of the HDAD film indicate its superior optical gain performance. The optical losses, a key determinant of ASE performance, were measured via the shifting excitation spot method. In this approach, a fixed‐length pump stripe is displaced from the sample edge, the ASE intensity follows the Lambert–Beer law: $I=I_{0}\;\exp\left(-\alpha x\right)$, with x being the unpumped distance between the stripe end and the sample edge, where $I_{0}$ is the initial intensity and α is the loss coefficient. The loss spectra (Supporting Information S1: Figure S3) decay exponentially, reflecting contributions from the absorption tail and scattering losses, yielding loss coefficients α of $2.18\;{cm}^{-1}$ (HDAD) and $2.69\;{cm}^{-1}$ (PA), further confirming the superior waveguiding performance of the HDAD films.

To gain deep insight into the ultrafast dynamics underlying the ASE of both perovskite films, we performed TA measurements. The GSB positions of the various n‐phases align well with the corresponding steady‐state absorption (Figure 2a–d). The PA film exhibits a phase distribution dominated by the n=2 phase; however, the HDAD film shows dominant contributions from both n=2 and n≥5 phases, indicating a more homogeneous phase distribution. As the time delay increases, the GSB intensity of the small‐n phases gradually decreases, while that corresponding to the 3D‐like large‐n phases (n≥5) continues to rise, verifying the energy funneling effect that energy is transferred from the small‐n phases to the high‐n phases for both quasi‐2D perovskites (Figure 2b–e). Figure 2c–f show the TA curves of the maximum ${\text{GSB}}_{n=2}$ and ${\text{GSB}}_{n\geq 5}$ for the PA and HDAD thin films, respectively. The inset shows the dynamic curve for ${\text{GSB}}_{n\geq 5}$ for the PA and HDAD thin films, respectively. The energy transfer efficiency in PA and HDAD films was calculated based on population evolution of the phase distribution, that is, the ratio between the GSB decrease of small‐n (n=2,3, and 4) phases and the GSB increase in large‐n (n≥5) phases. The HDAD film exhibits a higher energy transfer efficiency (33.3%) compared to that of the PA film (24.3%), with an absolute difference of 9 percentage points (Supporting Information S1: Table S2). Both the decay time (0.31 ps) of the n=2 phase in the HDAD film and the rise time (0.51 ps) of the n=5 phase are shorter than the corresponding values measured for the PA film (0.51 and 0.58 ps, respectively). The short decay time of the n=2 donor phase and short rise time of the 3D‐like acceptor phase reveal faster energy transfer. This process facilitates radiative recombination and improves light‐emission performance by suppressing exciton quenching. The extended carrier lifetime is beneficial for reducing the ASE threshold [22].

**FIGURE 2:**
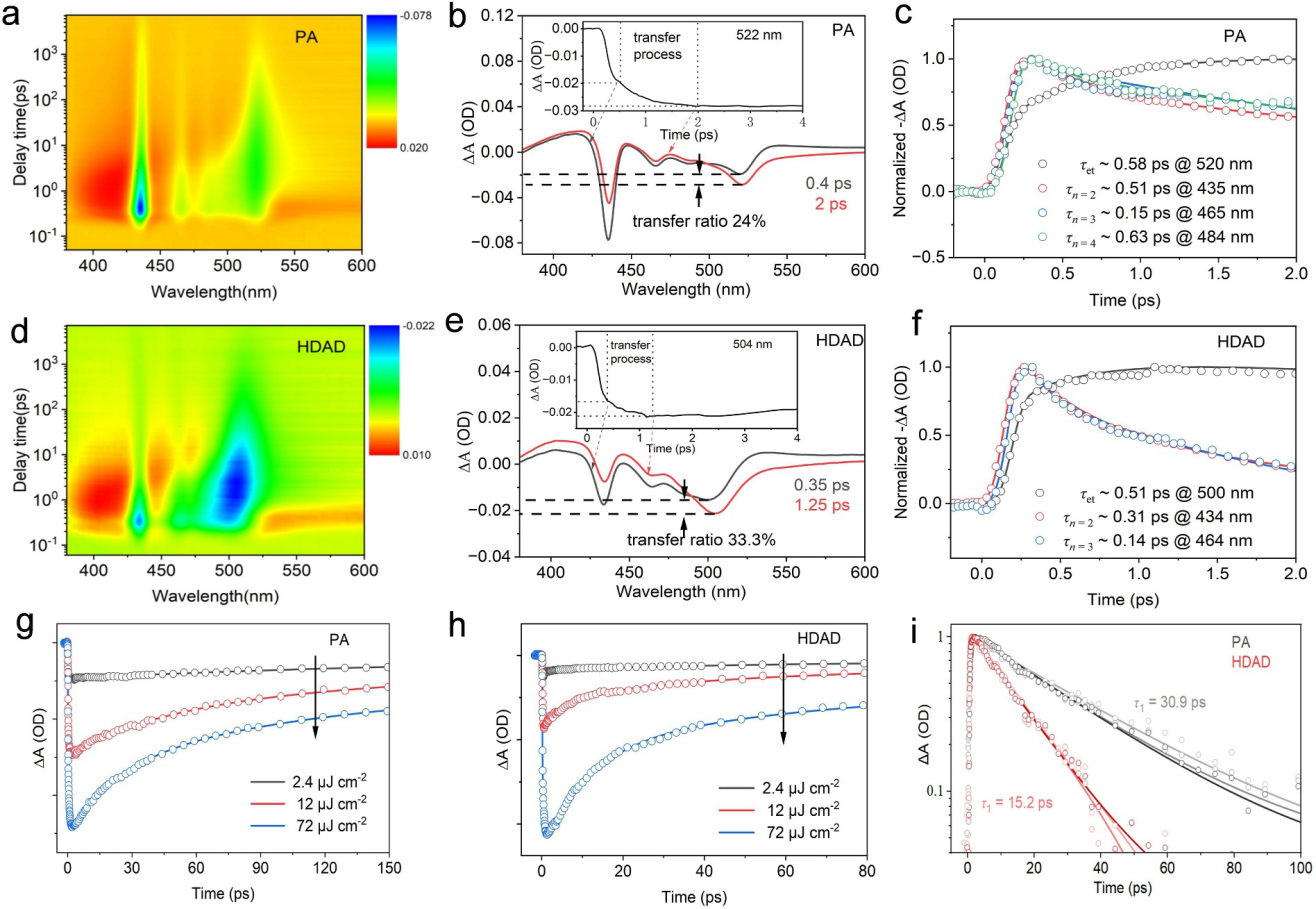
TA spectra and Auger recombination of the PA and HDAD thin films. (a, d) TA contour. (b, e) dynamic curves. (Insets: dynamic curves for ${GSB}_{n\geq 5}$). (c, f) TA spectra at selected delay times. (g, h, i) Auger recombination.

Auger recombination is a non‐negligible factor when examining the parameters that govern ASE thresholds in quasi‐2D perovskite thin films. Pump‐power‐dependent TA spectra reveal the presence of one fast‐rising component and one decay component at pump fluences of 2.6, 12.0, and 72.0 μJ ${\text{cm}}^{-2}$, respectively (Figure 2g,h). Notably, the fast decay component emerges and grows increasingly prominent as the pump power increases, which constitutes a typical signature of biexciton Auger recombination, rooted in the double degeneracy of the valence band edge transitions in lead halide perovskites [23]. The biexciton Auger recombination dynamics were derived via a pairwise subtraction procedure under three different pumping fluences, and subsequently fitted with a single‐exponential function (Figure 2i). The Auger recombination lifetime of the PA film (30.9 ps) is approximately twice as long as that of the HDAD film (15.2 ps), indicating that Auger recombination is more pronounced in the HDAD film, a trend that contrasts with the observed energy transfer efficiency. Compared with the PA sample, the HDAD‐HBr film features fewer low‐n impurity phases, higher PLQY, and reduced nonradiative trap losses, which can collectively increase the density of gain carriers [16, 24]. Although the HDAD‐HBr film exhibits faster Auger recombination, the increased population of radiative excitons outweighs the associated Auger losses. Furthermore, the smoother and more uniform morphology of the HDAD‐HBr film, as confirmed by scanning electron microscopy (SEM) images (Supporting Information S1: Figure S4), reduces optical scattering losses, thereby offsetting its stronger Auger recombination.

A trace amount of HBr acid was introduced into the precursor solution to improve phase purity during nucleation and growth processes. The HBr‐treated films exhibit much smoother and more homogeneous surfaces with notably fewer pinholes compared to the pristine film (Supporting Information S1: Figure S4). This morphological improvement originates from the bromide‐ion filling effect. Both films exhibit diffraction peaks only around 15° and 30° (Figure 3a). Besides, the FWHM of the main diffraction peak for the (100) plane decreased from 0.53° for the HBr‐treated PA film (PA‐HBr) to 0.42° for the HDAD‐HBr film, and that for the (200) plane decreased from 0.59° to 0.47°, indicating a larger grain size, fewer defects, higher phase purity, and enhanced crystallographic orientation after the addition of HBr [25]. The HDAD‐HBr film shows significantly enhanced absorption peaks for small‐n phases (n=2, 3) (Figure 3b). The enhanced PL intensity further confirms effective suppression of nonradiative recombination and passivation of interfacial defects. A redshift of the absorption tail by 4 nm (506 nm for the HDAD‐HBr film vs. 502 nm for the PA‐HBr film indicates a larger average 〈n〉 value in the HDAD‐HBr film. The increased PL intensity and the observed redshift are attributed to the filling effect of Br^−^ ions [26]. In the TRPL decay profiles, the HDAD‐HBr film exhibits an average decay lifetime of 17 ns, approximately 1.5 times longer than that of the PA‐HBr film (10.2 ns), indicating suppressed defect‐assisted nonradiative recombination (Figure 3c). This improvement is ascribed to the Br‐rich environment reducing Br vacancies [27].

**FIGURE 3:**
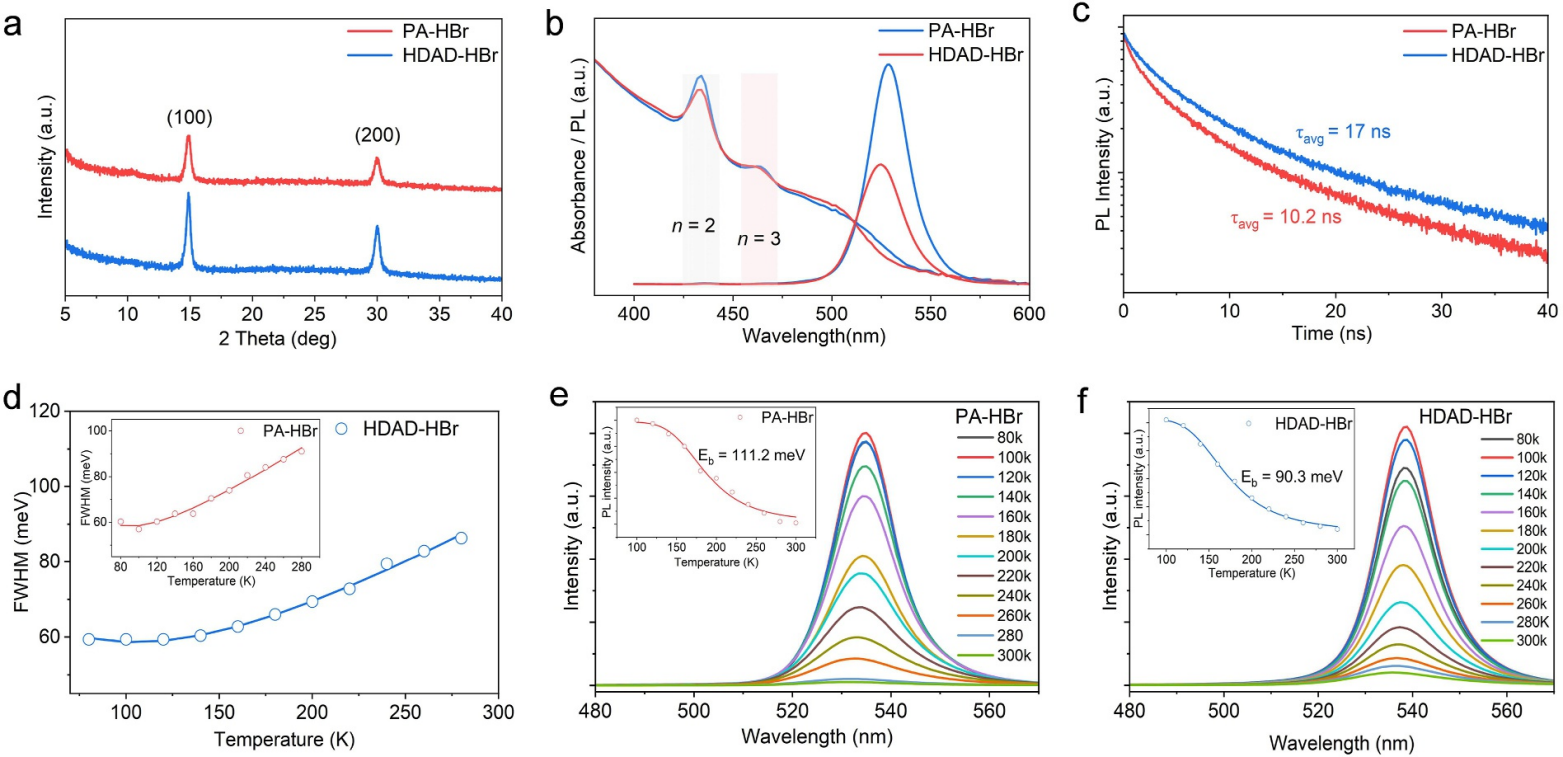
Structure and optical performance of the PA‐HBr and HDAD‐HBr thin films. (a) XRD patterns. (b) Absorption and PL spectra. (c) TRPL decays of the films. (d) Temperature‐dependent PL FWHM and the corresponding fitting for HDAD film and PA film (inset) with HBr treatment, respectively. (e, f) Temperature‐dependent integrated PL intensity with HBr‐treated films, respectively. Insets: temperature‐dependent integrated PL intensities and corresponding fit curves.

The carrier–phonon coupling strength was evaluated by fitting the temperature‐dependent PL spectra of the two films (Figure 3d). The fitting formula is $\Gamma \left(T\right)=\Gamma_{0}+\Gamma_{LO}=\Gamma_{0}+\gamma_{LO}/\left(\exp\left(E_{LO}/k_{B}T\right)-1\right)$, where $\Gamma_{0}$ is a temperature‐independent constant related to scattering from disorder and/or crystallographic imperfections, $\Gamma_{LO}$ represents the longitudinal optical phonon (Fröhlich) interactions, $\gamma_{LO}$ is the carrier–phonon coupling strength, and Γ(T) denotes the FWHM of the PL spectrum at the corresponding temperature [28]. The fitting results are summarized in Supporting Information S1: Table S3. The $\Gamma_{0}$ values for the PA‐HBr film and HDAD‐HBr films are 129.2 and 74.4 meV, respectively. This indicates that the incorporation of HBr more effectively suppresses the scattering phenomena in the HDAD‐film caused by defects or nonuniformities. The extracted carrier–phonon coupling strength $\gamma_{LO}$ is 243.3 and 189.3 meV for the PA‐HBr film and HDAD‐HBr film, respectively. The suppressed $\gamma_{LO}$ value for the HDAD‐HBr film would slow the carrier cooling rate and extend radiative recombination lifetime. The strong coupling constant implies that more excitons undergo localization via lattice thermal fluctuations in the pristine film [29]. The carrier–phonon coupling is detrimental to laser emission [30]. Figure 3e,f display the temperature‐dependent PL peak intensities I(T) under 450 nm excitation, where the PL intensity increases with decreasing temperature. The data are fitted with a formula, $I\left(T\right)=I_{0}/1+A\;\exp\left(-E_{b}/k_{B}T\right)$, where $k_{B}$ is the Boltzmann constant, $E_{b}$ is the exciton binding energy, and $I_{0}$ and A are constants [31]. The exciton binding energy is determined to be 111.2 and 90.3 meV for the PA‐HBr film and HDAD‐HBr film, respectively. The addition of HBr reduced the exciton binding energy and suppressed the Auger recombination rate of both the PA film and the HDAD film. However, the improvement in the HDAD film was significantly greater than that in the PA film (Supporting Information S1: Figure S5 and Table S3).

The TA spectra demonstrate that the growth of the small‐n phases (n=2, 3) is promoted (Figure 4a,b). The HDAD‐HBr film exhibits an energy transfer efficiency of 34.2%, higher than that of the pristine films (33.3%) (Supporting Information S1: Table S2). The Auger lifetime of the HDAD‐HBr film is 17.3 ps, longer than that of HDAD film (15.2 ps) (Figure 4c). The Auger recombination rate is proportional to the exciton binding energy $\left(E_{b}\right)$ [32]. The HDAD‐HBr film achieves an ultralow threshold of 5.5 μJ ${\text{cm}}^{-2}$ (Figure 4d).

**FIGURE 4:**
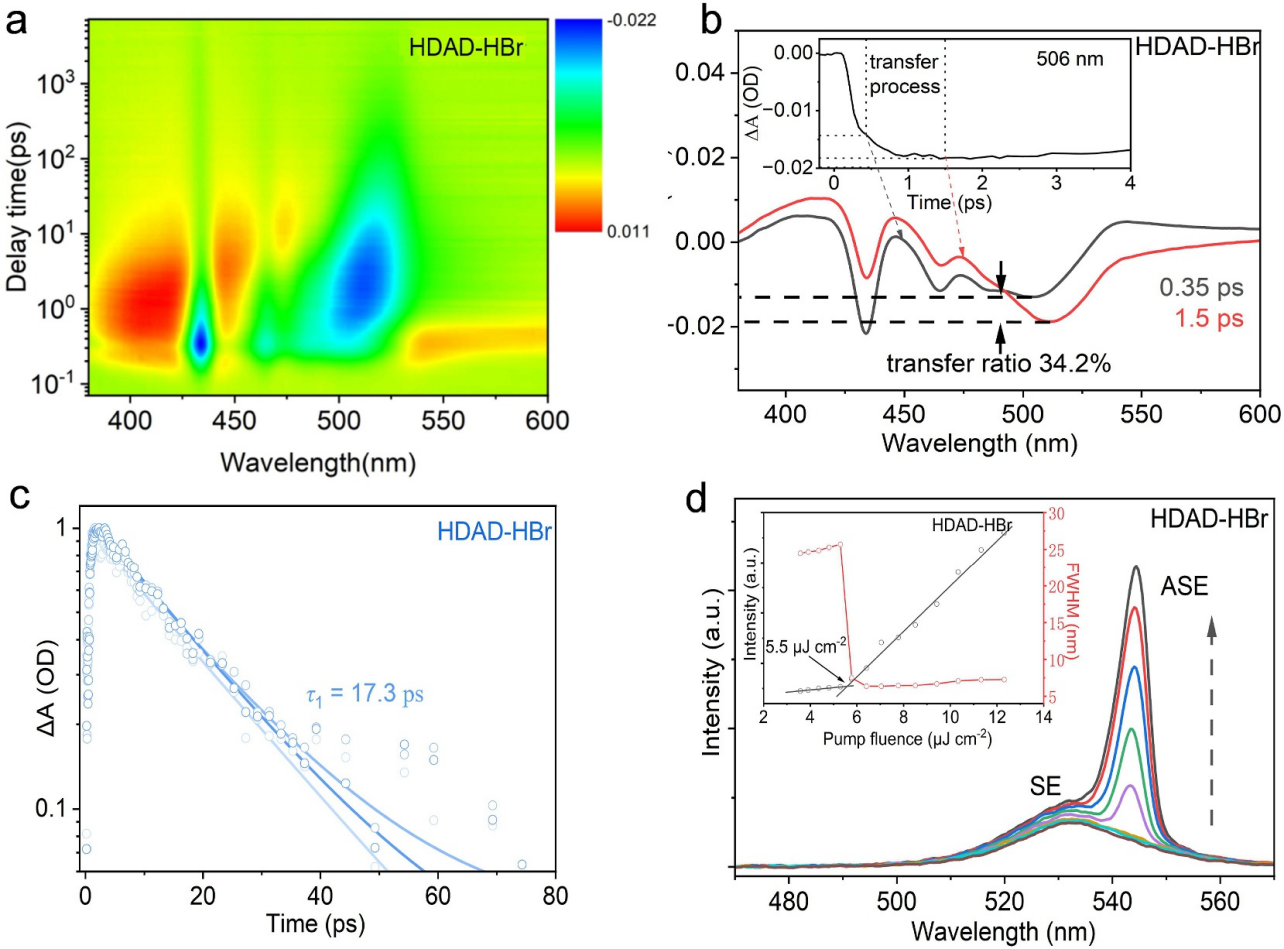
TA spectra and ASE properties of the HDAD‐HBr thin film. (a) TA colormap. (b) TA spectra at selected delay times. Inset: the transfer process in the carrier dynamics of perovskite thin films. (c) Auger recombination dynamics of the film. (d) ASE threshold of the film. Inset: PL intensities and FWHMs as functions of pump intensities.

The distributed Bragg reflector (DBR) mirrors exhibit a high reflectivity of 99.8% around 542 nm (Figure 5a). An optically pumped green laser was fabricated by sandwiching the HBr‐treated (HDAD) (${\text{FA}}_{0.8}{\text{Cs}}_{0.2}$)_3_ ${\text{Pb}}_{4}{\text{Br}}_{13}$ perovskite between two DBR mirrors, as illustrated in Figure 5b,c presents PL spectra collected at room temperature under varied pump fluences ranging from 3.78 μJ cm^−2^ to 56.24 μJ cm^−2^. A single‐mode emission centered at 542 nm with an FWHM of approximately 0.42 nm is observed (Figure 5d). The inset in Figure 5d shows the light output and FWHM as a function of pump fluence. The abrupt slope change (from 2.0 to 4.7) in the PL intensity versus pump fluence curve marks the transition from SE to the ASE regime. The ASE threshold of the HDAD‐HBr‐based device was determined to be $16.5\;\mu J\;cm^{-2}$ via pump‐dependent PL measurements, and this value is comparable to previously reported results summarized in Supporting Information S1: Table S4. These results demonstrate that HDAD‐HBr‐based quasi‐2D films are promising gain media for laser applications.

**FIGURE 5:**
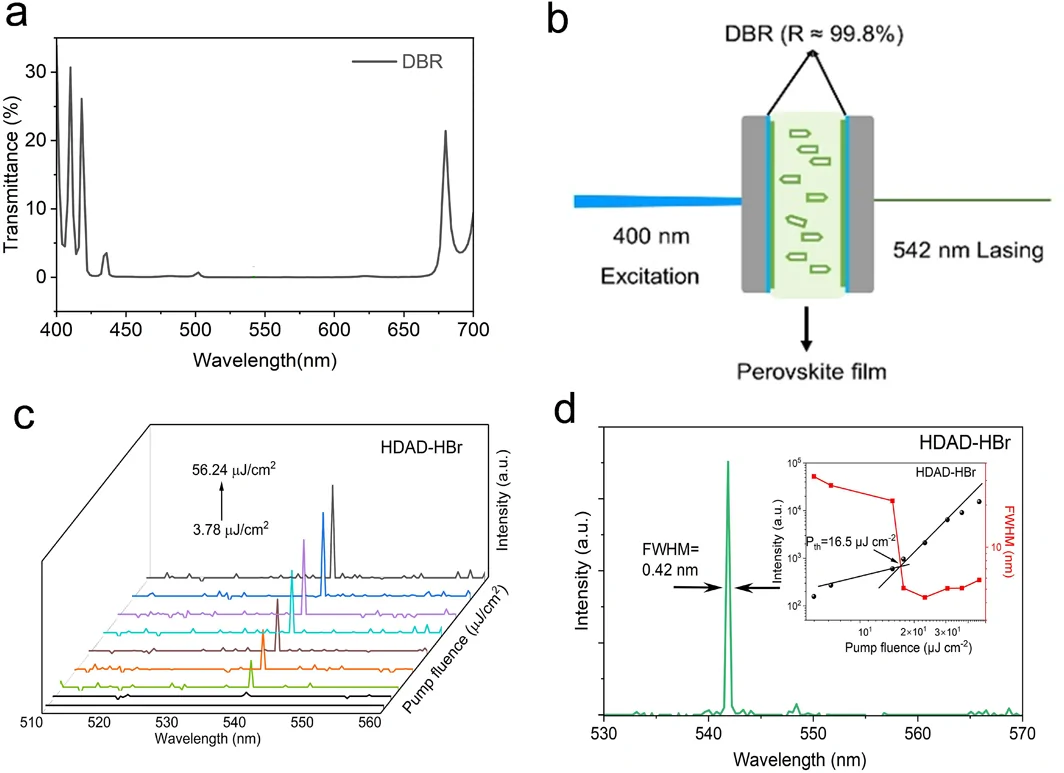
Lasing performance. (a) Transmission spectrum of the DBR mirror. (b) Schematic of the VCSEL structure. (c) Evolution of PL spectra under different pump fluences. (d) Lasing emission spectrum of the device. Inset: Light output and FWHM versus pump fluence, indicating a threshold of $16.5\;\mu J\;cm^{-2}$.

## 3 Conclusion

In summary, carrier dynamics and ASE performance are investigated by comparing the phase distribution, defect density, and Auger recombination of PA and HDAD quasi‐2D perovskite films. The HDAD‐HBr exhibits a lower ASE threshold of 5.5 μJ ${\text{cm}}^{-2}$ due to suppressed Auger recombination and exciton–phonon coupling. Furthermore, a single‐mode vertical‐cavity laser with a linewidth of 0.42 nm and a threshold of 16.5 μJ ${\text{cm}}^{-2}$ was demonstrated. These results present an effective strategy for achieving low‐threshold quasi‐2D perovskite lasers through precise modulation of phase distribution and crystal quality.

## 4 Experimental Section

### 4.1 Materials

Hexane‐1,6‐diammonium dibromide [$C_{6}H_{18}N_{2}Br_{2}$, $HDABr_{2}$] (99.5%, Xi'an Polymer Light Technology Corp. Catalog No.301090), propylammonium bromide [$C_{3}H_{10}BrN$, (PA)Br] (99.5%, Xi'an Polymer Light Technology Corp. Catalog No.301038), formamidinium bromide [$CH_{5}N_{2}Br$, (FA)Br] (99.9%, Xi'an Polymer Light Technology Corp., Catalog No.301011), lead (II) bromide $\left(PbBr_{2}\right)$ (99.99%, Xi'an Polymer Light Technology Corp. Catalog No.301008), and cesium bromide (CsBr) (99.9%, Xi'an Polymer Light Technology Corp. Catalog No.301046) were purchased from Xi'an Polymer Light Technology Corp. Dimethyl sulfoxide [$C_{2}H_{6}OS$, DMSO] (99.5%, Aladdin, Catalog No. D670381), and aqueous HBr solution (98%, Aladdin, Catalog No.P342998) were purchased from Aladdin Chemical Reagents Co. Ltd. All chemicals were used as received without further purification.

### 4.2 Film Preparation

#### 4.2.1 Preparation of (PA)_2_ (${\text{FA}}_{0.8}{\text{Cs}}_{0.2}$)_3_ ${\text{Pb}}_{4}{\text{Br}}_{13}$ and (HDAD) (${\text{FA}}_{0.8}{\text{Cs}}_{0.2}$)_3_ ${\text{Pb}}_{4}{\text{Br}}_{13}$

0.014 g of PABr [0.0139 g of (HDAD)Br_2_], 0.0064 g of CsBr, 0.0149 g of FABr, and 0.0734 g of ${\text{PbBr}}_{2}$ were dissolved in 1 mL of DMSO to prepare the perovskite precursor solution. The solution was stirred at room temperature for approximately 2 hours and then transferred to a glove box. Glass substrates were cleaned with plasma for more than 15 minutes. 30 μL of the precursor solution was dropped onto the glass substrate and spin‐coated at 4000 rpm for 50 s, then transferred to an annealing stage for annealing at 90 °C for 15 minutes. (PA)_2_ (${\text{FA}}_{0.8}{\text{Cs}}_{0.2}$)_3_ ${\text{Pb}}_{4}{\text{Br}}_{13}$ and (HDAD) (${\text{FA}}_{0.8}{\text{Cs}}_{0.2}$)_3_ ${\text{Pb}}_{4}{\text{Br}}_{13}$ films treated with HBr were prepared by adding 2 μL of HBr to the precursor solution.

#### 4.2.2 Single‐Mode Laser Devices Based on HBr‐Treated (HDAD) (${\text{FA}}_{0.8}{\text{Cs}}_{0.2}$)_3_ ${\text{Pb}}_{4}{\text{Br}}_{13}$

The distributed Bragg reflector (DBR) substrates were sequentially cleaned with absolute alcohol and deionized water, followed by a plasma cleaning treatment for 15 min. It was then transferred to an argon‐filled glove box, where 30 μL precursor solution was drop‐cast onto the substrate, spin‐coated at 4000 rpm for 50 s, and annealed at 90 °C for 15 minutes. Finally, the two DBR layers were bonded together with an adhesive.

### 4.3 Characterizations

#### 4.3.1 XRD (SAXRD), SEM, Steady‐State Absorption, PL, PLQY, and TRPL Measurements

The crystal structures of (PA)_2_ (${\text{FA}}_{0.8}{\text{Cs}}_{0.2}$)_3_ ${\text{Pb}}_{4}{\text{Br}}_{13}$ and (HDAD) (${\text{FA}}_{0.8}{\text{Cs}}_{0.2}$)_3_ ${\text{Pb}}_{4}{\text{Br}}_{13}$ films were characterized by using XRD, employing a scanning rate of 0.05° per second in a 2θ range from 2° to 50° (from 0.5° to 10°), using an X‐ray diffractometer with Cu Kα radiation (D8 Discover, Bruker). The morphology of the films was analyzed using a field‐emission SEM (S‐4800, Hitachi). The absorption spectra of films were measured by UV–vis spectrophotometer (Cary‐60, Agilent). The PL spectra were measured by a spectrofluorometer (FluoroMax Plus, Horiba). PLQY measurements were performed using an integrating sphere (Horiba Quanta‐ϕ) coupled to a FluoroMax Plus fluorescence spectrometer, with an excitation wavelength of 375 nm. The PLQY values were calculated using standard methods with a blank reference, defined as the ratio of the number of fluorescence photons emitted after sample absorption to the number of absorbed excitation photons. The formula is expressed as $Y=k/\left(k+\sum K\right)$, where Y is the PLQY, k is the fluorescence emission rate, and ∑K is the sum of the rates of various nonradiative decay processes. Evidently, Y<1. A higher PLQY value indicates fewer nonradiative recombinations in the sample and thus better performance. Each sample was measured at least three times at different positions, the reported values are averages with a standard deviation of approximately ±2%. For the TRPL measurements, samples were excited by a pulsed laser at 375 nm (> 28 pJ/pulse), using the integrated fluorescence spectrometer (FluoroMax Plus, Horiba) with a time‐correlated single‐photon counting (TCSPC) system. The temporal resolution of TRPL spectra is ∼25 ps.

#### 4.3.2 ASE and Lasing Measurements

For the ASE measurements, a portion of the fundamental 800 nm laser pulse (35 fs, 1 kHz, Coherent) is frequency‐doubled to 400 nm pump light by passing through a type‐I barium borate (BBO) crystal cut at 29.2°. The laser excitation wavelength is 400 nm (single photon), and the excitation spot diameter is 3 mm. The excitation light is introduced vertically into the quasi‐2D DJ film through a filter, and the emission from the film edge is collected vertically and detected with a fiber optic spectrometer (USB4000‐UV‐VIS, Ocean Optics).

#### 4.3.3 Ultrafast TA Measurements

The femtosecond transient absorption (TA) measurements were performed on a pump–probe system (Helios, Ultrafast Systems). White‐light continuum probe pulses in the range of 390–600 nm were generated by focusing a small fraction of the fundamental 800 nm laser pulses (800 nm, 35 fs, 7 mJ/pulse, 1 kHz repetition rate, and Coherent) onto a calcium fluoride crystal. For the TA measurements, the pump pulses centered at 365 nm were produced by an optical parametric amplifier (TOPAS‐800‐fs and Light‐Conversion), and the probe polarization was set at the magic angle. For the measurements of ASE and gain coefficient, the samples were excited by 400 nm pulses obtained from frequency‐doubling of the fundamental 800 nm beam. The instrument response function was determined to be ∼100 fs by a routine cross‐correlation procedure. The kinetic traces were fitted with a multiexponential function: $\Delta A\left(t\right)=a_{1}\;\exp\left(-t/\tau_{1}\right)+a_{2}\;\exp\left(-t/\tau_{2}\right)-c_{1}\;\exp\left(-t/\tau_{\text{et}}\right)$, where $a_{1}$, $a_{2}$, and $c_{1}$ are amplitudes, $\tau_{1}$ and $\tau_{2}$ are decay time constants, and $\tau_{\text{et}}$ is the formation time constant.

#### 4.3.4 The Optical Gain

The optical gain was measured using the optical setup illustrated in Supporting Information S1: Figure S6. A laser source operating at a central wavelength of 400 nm, with a repetition rate of 1 kHz and a pulse duration of 35 fs, was employed as the excitation source. The 400 nm beam was generated by frequency doubling the fundamental 800 nm output using a β‐barium borate (BBO) crystal. To prevent the 800 nm residual light from generating excess free carriers that could interfere with excitonic lasing, a short‐pass filter was used to block the fundamental wavelength. The spot size on the sample was adjusted using a convex lens, and a continuously variable neutral density filter controlled the pump fluence to characterize the ASE and lasing thresholds of the quasi‐2D perovskite films as a function of excitation energy density.

#### 4.3.5 The Loss Coefficient

The waveguide losses were characterized by conducting experiments to measure the optical loss coefficient. In these experiments, a pump stripe of fixed length was gradually displaced from the sample edge. Assuming that the amplified spontaneous emission (ASE) originating from the end of the excitation stripe remains constant, the emission collected from the sample edge should decrease as the stripe is displaced due to waveguide losses (absorption and scattering), following the Lambert–Beer law: $I=I_{0}e^{-\alpha x}$, where x is the unpumped distance between the pump stripe end and the sample edge. The loss measurements were performed by mounting the sample on a micrometer stage, and all measurements were conducted at least three times at each pump intensity to obtain an averaged value.

#### 4.3.6 Temperature‐dependent Photoluminescence (PL) Spectroscopy

The thermostat device was purchased from LNC‐W, with liquid nitrogen serving as the cooling source. The temperature control range is between 78 and 325 K, with a precision of ±50 mK. Before starting the cooling process with the test sample in place, it is important to evacuate the chamber to a vacuum level for at least 20 minutes. If the evacuation is not thorough enough, water droplets may form on the window, affecting the experiment. The pressure must reach at least $4\times 10^{-1}$ Pa before liquid nitrogen can be introduced. Care should also be taken with the amount of liquid nitrogen used: approximately 30 mL per session. This amount should be added in three portions: once to bring the temperature down to 200 K, and once more to lower it to 100 K. After reaching 80 K, wait for about 10 minutes for stabilization before adding another 10 mL of liquid nitrogen for better temperature control, after which the heating process can begin. Depending on your needs, you can measure data every 20 K intervals. It is crucial to allow each temperature to stabilize for at least 3 minutes before taking readings to avoid inaccurate results due to rapid temperature changes. Finally, wait at least half an hour after completing the measurements for the entire system to return to room temperature before disassembling the equipment and replacing the sample.

## Author Contributions

**Chaochao Qin:** conceptualization, methodology, supervision, resources, project administration, formal analysis, funding acquisition, writing – review and editing. **Xinyu Zhang:** investigation, writing – original draft, writing – review and editing. **Minghuan Cui:** software, funding acquisition. **Haiying Wang:** writing – review and editing. **Pan Song:** writing – review and editing. **Jian Song:** writing – review and editing. **Shuhong Ma:** writing – review and editing. **Zhaoyong Jiao:** writing – review and editing. **Guangrui Jia:** writing – review and editing. **Yuhai Jiang:** project administration, visualization. **Zhongpo Zhou:** writing – review and editing, validation, supervision, conceptualization, funding acquisition, visualization, formal analysis, data curation.

## Supporting information


Supporting Information S1

## Data Availability

The data that support the findings of this study are available from the corresponding author upon reasonable request.
