# Supplementary material for: Amplified Spontaneous Emission and Carrier Dynamics in Ruddlesden–Popper and Dion–Jacobson Quasi‐2D Perovskite
Source: Nanophotonics. 2026 Nov 10;15(21):e70335. doi: 10.1002/nap2.70335 (PMC13752555; doi:10.1002/nap2.70335)
Supplement: Supplementary file 1 [file NAP2-15-e70335-s001.docx]

**Supporting information**

**Amplified spontaneous emission and carrier dynamics in Ruddlesden-Popper and Dion-Jacobson** **quasi-2D perovskites**

Chaochao Qin^1^, Xinyu Zhang^1^, Minghuan Cui^1^, Haiying Wang^1^, Pan Song^1^, Jian Song^1^, Shuhong Ma^1^, Zhaoyong Jiao^1^, Guangrui Jia^1^, Yuhai Jiang^1^, Zhongpo Zhou^1,[1]a)^

^1^Henan Province Engineering Research Center of Optoelectronic Technology and Advanced Manufacturing, and School of Physics, Henan Normal University, Xinxiang, 453007, China

Figure S1. SAXRD of quasi-2D perovskites.

Figure S2. PLQY of PA film, HDAD film, and films treated with HBr acid.

Figure S3. Optical loss coefficient of the thin film. a) PA. b) HDAD.

Figure S4. SEM images of the films. a) PA. b) HDAD. c) PA-HBr. d) HDAD-HBr.

Figure S5. a) Temperature-dependent PL FWHM. b) Fitted curves of the integrated PL intensity as a function of temperature for perovskite films of the pristine PA and HDAD films.

Figure S6. Schematic diagram of the optical system for gain and lasing-emission measurements.

**Table S1**. TRPL Lifetime of the quasi-2D perovskite thin films.

| Sample | A_1_ | *τ*_1_  (ns) | A_2_ | *τ*_2_  (ns) | *τ*_avg_  (ns) |
| --- | --- | --- | --- | --- | --- |
| PA | 0.54 | 0.82 | 0.45 | 8.41 | 4.30 |
| HDAD | 0.60 | 1.9 | 0.39 | 12.3 | 6.0 |
| PA-HBr | 0.65 | 3.43 | 0.34 | 23.48 | 10.2 |
| HDAD-HBr | 0.65 | 3.7 | 0.34 | 42.19 | 17 |

**Table S2**. Dynamic changes and transfer ratio of the TA spectra for thin films.

| Sample | ΔA_n=2_  (mOD) | ΔA_n=3_  (mOD) | ΔA_n=4_  (mOD) | ΔA_n≥5_  (mOD) | Transfer  ratio (%) |
| --- | --- | --- | --- | --- | --- |
| PA | -33 | -4 | -4 | 10 | 24.3 |
| HDAD | -10 | -5 | -3 | 6 | 33.3 |
| HDAD-HBr | -11 | -6 | -3.3 | 7 | 34.2 |

**Table S3**. Parameters extracted from the temperature-dependent PL spectra of films without and with the addition of HBr.

| **Sample** | $\boldsymbol{E}_{\boldsymbol{b}}$ **(meV)** | ***Γ*_0_ (meV)** | ***γ*_LO_ (meV)** | ***E*_LO_ (meV)** |
| --- | --- | --- | --- | --- |
| PA | 125.6 | 148.5 | 289.7 | 8.2 |
| HDAD | 99.7 | 96.3 | 212.5 | 22.6 |
| PA-HBr | 111.2 | 129.2 | 243.3 | 10.3 |
| HDAD-HBr | 90.3 | 74.4 | 189.3 | 31.1 |

**Table S4.** Summary of ASE properties of quasi-2D perovskite films reported in the literature.

| **Gain material** | **Pump source** | **solvent** | **ASE threshold (µJ cm^−2^)** | **Ref.** |
| --- | --- | --- | --- | --- |
| PEABr:FAPbBr_3_ | 150fs, 400nm | DMSO | 51.1 | ^1^ |
| PEABr:CsPbBr_3_ | 120fs, 400nm | DMSO | 11.7 | ^2^ |
| PEABr:CsPbBr_3_ | 230fs, 343nm | DMSO | 12.5 | ^3^ |
| PEABr:CsPbBr_3_ | 35fs, 400nm | DMSO | 2.6 | ^4^ |
| NMA_0.8_:Cs_0.87_(FAMA)_0.13_PbBr_3_ | 80fs, 400nm | DMSO | 3.8 | ^5^ |
| OABr:CsPbBr_3_ | 1ns, 355nm | DMSO | 14.9 | ^6^ |
| PDMABr_2_:MAPbBr_3_ | 35fs, 400nm | DMSO | 17.1 | ^7^ |
| CsPbBr₃:TPPO (Thermal Evaporation) | 35fs, 400nm | Thermal Evaporation | 6.0 | ^8^ |
| HBr: HDADBr:PABr_2_ | 35fs, 400nm | DMSO | 16.5 | This work |

**References**

1 L. Lei, D. Seyitliyev, S. Stuard, J. Mendes, Q. Dong, X. Fu, Y. Chen, S. He, X. Yi, L. Zhu, C-H. Chang, H. Ade, K. Gundogdu, and F. So, [Adv. Mater.](https://onlinelibrary.wiley.com/doi/full/10.1002/adma.201906571) **32**, 1906571 (2020).
2 J. Li, L. Zhang, Z. Chu, C. Dong, J. Jiang, Z. Yin, J. You, J. Wu, W. Lan, and X. Zhang, [Adv. Optical Mater.](https://onlinelibrary.wiley.com/doi/full/10.1002/adom.202102563) **10**, 2102563 (2022).
3 G. Jin, T. Liu, Y. Li, J. Zhou, D. Zhang, P. Pang, Z. Ye, Z. Xing, G. Xing, J. Chen, and D. Ma, [Nanoscale.](https://www.x-mol.com/paperRedirect/1479173999599702016) **14**, 919 (2022).
4 M. Cui, C. Qin, Y. Jiang, M. Yuan, L. Xu, D. Song, Y. Jiang, and Y. Liu, [J. Phys. Chem. Lett.](https://pubs.acs.org/doi/full/10.1021/acs.jpclett.0c01852) **11**, 5734 (2020).
5 M. Li, Q. Shang, C. Li, S. Li, Y. Liang, W. Yu, C. Wu, L. Zhao, Y. Zhong, W. Du, X. Wu, Z. Jia, Y. Gao, H. Chen, X. Liu, S. Guo, Q. Liao, G. Xing, L. Xiao, and Q. Zhang, [Adv. Funct. Mater.](https://onlinelibrary.wiley.com/doi/full/10.1002/adfm.202102210) **31**, 2102210 (2021).
6 J. Li, W. Zhou, L. Jiang, Z. Fang, Z. Yang, C. Lin, X. Xu, Z. Ye, H. Zhu, and H. He, [J. Mater. Chem. C.](https://pubs.rsc.org/en/content/articlelanding/2019/tc/c9tc05090f) **7**, 15350 (2019).
7 C. Qin, Q. Huang, S. Zhang, J. Song, Z. Jiao, S. Ma, S. Zheng, P. Song, H. Wang, J. Zhang, G. Jia, Y. Jiang, and Z. Zhou, Appl. Phys. Lett. 124(8), 081103 (2024).
8 S. Huang, Z. Shen, Y. Liao, Z. Liu, Z. Hu, Q. Li, Z. Zhang, S. Dong, J. Luo, J. Du, J. Tang, Y. Leng,  Adv. Mater. 2023, 35, 2306102.

1. ^a)^ Author to whom correspondence should be addressed: zpzhou@htu.edu.cn.
